# Large-scale reduction of tyrosine kinase activities in human monocytes stimulated *in vitro* with *N*. *meningitidis*

**DOI:** 10.1371/journal.pone.0181912

**Published:** 2018-01-19

**Authors:** Unni Gopinathan, Kathrine Røe Redalen, Anne-Marie Trøseid, Peter Kierulf, Petter Brandtzaeg, Anne Hansen Ree, Jens Petter Berg, Reidun Øvstebø

**Affiliations:** 1 Blood Cell Research Group, Section for Research, Department of Medical Biochemistry, Oslo University Hospital, Oslo, Norway; 2 Institute of Clinical Medicine, Faculty of Medicine, University of Oslo, Oslo, Norway; 3 Department of Oncology, Akershus University Hospital, Lørenskog, Norway; 4 Department of Pediatrics, Oslo University Hospital, Oslo, Norway; University of Kansas Medical Center, UNITED STATES

## Abstract

*N*. *meningitidis* induces extensive gene expression changes in human monocytes, suggesting that complex networks of signaling pathways are activated during meningococcal sepsis. These effects are modulated by the anti-inflammatory cytokine interleukin-10 (IL-10). To further study changes in signal transduction suggested by mRNA data, we used kinase substrate arrays to identify composite kinase activities induced by lysates from a primary human monocyte model system. Cell lysates were prepared from monocytes treated with the following experimental conditions: 10^6^ *N*. *meningitidis*/mL, 25 ng/mL IL-10, 10^6^ *N*. *meningitidis*/mL in combination with 25 ng/mL IL-10, and vehicle. Lysates were subjected to kinase activity profiling with Tyrosine Kinase PamChip® arrays containing 144 kinase peptide substrates. In our experimental model, we were not able to detect a statistically significant large-scale change in *ex vivo* array peptide phosphorylation by lysates from monocytes treated for 15 minutes. Targets of the IL-10 anti-inflammatory response were not identified. A profound inhibition of array peptide phosphorylation by monocytes treated for 60 minutes was identified, suggesting low activity of a large number of kinases associated with different signaling pathways and immune cell functions, including STAT3 activity, Nf-κB and VEGF signaling, and PTEN signaling activity. The peptide representing ZBTB16, which was reduced in phosphorylation by lysates from all three experimental conditions, was in Ingenuity Pathway Analysis identified to be linked to reduced cytokine release and mRNA levels of tumor necrosis factor (TNF), IL-6, and CXCL10. Further studies should investigate changes in tyrosine kinase-mediated signal transduction in human immune cells, in order to evaluate the potential clinical application of kinome profiling in the study of systemic inflammatory responses to pathogens.

## Introduction

Meningococcal sepsis is an overwhelming form of the sepsis syndrome which may cause mortality within 12–24 hours in previously healthy children and adults [1]. The causative infectious agent is *N*. *meningitidis*—an obligate human pathogen. The rapidly evolving course of the disease with septic shock, multi-organ failure, coagulopathy, and mortality is associated with the high levels of meningococci and lipopolysaccharide (LPS, endotoxin) in the blood [2–4].

Previous studies have identified that clinically relevant concentrations of *N*. *meningitidis* may alter expression of over 4600 genes (fold change (FC) ≥2.0, uncorrected *P*<0.05) in human monocytes [5]. Extensive gene expression changes in monocytes are also induced by plasma from patients with meningococcal sepsis [6]. These gene expression changes are modulated by the anti-inflammatory cytokine interleukin-10 (IL-10), which is present in large quantities in plasma from patients with meningococcal sepsis [7–9]. Overall, the gene expression results suggest that complex networks of signaling pathways are activated and inhibited during meningococcal sepsis. This is also in line with a number of other studies over the past decade investigating transcriptomic changes in immune cells from patients with various forms of sepsis [10–12]. However, one limitation when interpreting mRNA expression findings is that due to variation in translational activity and post-translational modifications, these may suggest, but not necessarily accurately reflect the cellular phenotypes involved. Studies using proteomic methods should therefore follow up transcriptomic studies.

Studying the activity of protein kinases has been suggested as one strategy for gaining insights into signal transduction affecting various cellular processes including metabolism, transcription, cell cycle progression, cytoskeletal rearrangement and cell movement, apoptosis, and differentiation [13]. Protein serine, threonine or tyrosine kinases are a family of enzymes mediating signal transduction by catalyzing the transfer of a γ phosphate group from ATP to a specific serine, threonine or tyrosine hydroxyl group in a target protein or peptide. It has been estimated that protein kinases modify one-third of the human proteome [13,14].

The new generation of kinase substrate arrays offers methods for parallel analysis of multiple kinase activities, also known as kinome profiling. Kinase substrate array technology may assay tissue samples for the activity of protein serine, threonine or tyrosine kinases. Until now, this method has predominantly been used to identify altered levels of kinase activities in cancer tissues, and to evaluate treatment response in cancer. Only a few studies, to our knowledge, have used kinome profiling to investigate changes in kinase activities in infectious diseases [15,16]. Tyrosine phosphorylation has for long been implicated in LPS-CD14-TLR4 signal transduction [17–19], which is the dominant pathway through which *N*. *meningitidis* induces pro-inflammatory activation in monocytes [20]. Kinase profiling may therefore represent a promising strategy for investigating the host response of human monocytes to *N*. *meningitidis*. In the present study, we investigated composite kinase signaling activities in an *in vitro* primary cell model of elutriated and cryopreserved human monocytes [21]. This model has previously been used to study monocyte activation by meningococci to elucidate pathophysiological mechanisms associated with inflammatory capacity and TLR4 activation [7,20], coagulation [22,23], and gene expression [5,6,24]. Our present aim was to identify activation or inhibition of tyrosine phosphorylation by lysates from human monocytes stimulated *in vitro* with *N*. *meningitidis* in the presence and absence of IL-10. The altered kinase activities were also compared with previously generated data on gene expression changes and cytokine release from human monocytes stimulated with *N*. *meningitidis* and IL-10.

## Material and methods

### Ethics approval and consent to participate

This study used human monocytes elutriated from heparinized whole blood collected from healthy donors. Participants have provided written consent to the blood bank of Oslo University Hospital for blood to be used for research purposes. The biological material was used in accordance with ethics approval from the Regional Medical Ethics Committee of Health Region I in Norway (Ethics approval no. 2011/1413, biobank material access number 908; Human monocytes and lymphocytes; Oslo University Hospital, Oslo, Norway).

### N. meningitidis

The reference strain *N*. *meningitidis* 44/76 (serogroup B:15:P1:7,16), originally isolated from a culture of blood from a Norwegian patient with lethal meningococcal sepsis, was provided by the Norwegian Institute of Public Health. The bacteria were heat-inactivated (56^o^ C, 30 min) for safety reasons and the number of bacteria was quantified as previously described [3].

### Recombinant IL-10

Stock solution (1μg/mL) of recombinant human IL-10 (denoted hereafter as IL-10; cat. no. 217-IL-025, R&D Systems, www.rndsystems.com) was reconstituted by adding 1 mL phosphate-buffered saline (PBS) to the lyophilized powder, and stored in working aliquots of 50 μL at –70°C.

### Lysis buffer

Lysis buffer consisted of 980 μL Mammalian Protein Extraction Reagent (cat. no. 78503, ThermoFisher Scientific, www.thermofisher.com), 10 μL Halt Protease Inhibitor Cocktail (cat. no. 78415, Thermo Scientific), and 10 μL Halt Phosphatase Inhibitor Cocktail (cat. no. 78420, Thermo Scientific). The buffer was stored at -20°C prior to use.

### Primary human monocyte model system

Elutriated (>90% purity), cryopreserved human monocytes [21] from eight consenting, healthy donors were thawed and suspended in 5% (vol/vol) fetal calf serum (FCS) in RPMI 1640 containing 2% (vol/vol) penicillin-streptomycin. Monocytes were seeded (2 x 10^6^ monocytes suspended in 1 mL 5% (vol/vol) FCS-RPMI) in Costar® 6-well plates (Costar® 3471). Total volume in each well was 2 mL and total number of cells in each well was 4 million. The monocytes were stimulated with the following experimental treatments: 10^6^ *N*. *meningitidis*/mL (“***Nm”***), 25 ng/mL IL-10 ***(“IL-10”*),** 10^6^ *N*. *meningitidis*/mL in combination with 25 ng/mL IL-10 *(****“Nm + IL-10”*),** and vehicle (unstimulated controls). Each experimental condition was run in duplicates to ensure sufficient protein concentration in the monocyte lysates. The plates were sealed off and incubated for 15 minutes and 1 hour (37°C, 5% CO_2_) before being subjected to cell lysate preparation as described below. With respect to viability, our research group has previously systematically investigated the viability of monocytes in culture—based on the absence of annexin V and PI—and have identified around 80% viability of monocytes incubated with wild-type *N*. *meningitidis* after 4 hours [25]. Similar levels of viability after similar incubation times have been observed in subsequent studies [5,23]. Loss of viable monocytes due to apoptosis and necrosis is shown to increase over time, with the main cell loss occurring within the first 24 h. Given that we only stimulated monocytes for 15 minutes and 60 minutes using experimental conditions similar to previous studies, we have assumed viability to be at least as high as observed in these previous studies [5,23,24].

Incubation times were determined after a pilot experiment stimulating monocytes from a single donor for 0, 30, 60, and 120 minutes, which together with previous studies of tyrosine phosphorylation in LPS-stimulated monocytes [17,18,26–28], suggested that tyrosine kinase activity in monocytes reaches a peak between 15 minutes and 60 minutes.

### Preparation of cell lysates for kinase activity analysis

After completion of incubation times as described above, a rubber policeman was used to loosen monocytes attached to the plate surface. The cell suspension was transferred to microcentrifuge tubes, and centrifuged (800g, 5 min, 4°C). Culture supernatants were separately stored for later measurement of tumor necrosis factor (TNF) in all donors apart from EL303 which was discarded by error. To each pellet, 500 μL ice cold PBS was added, and suspended pellets corresponding to the same experimental condition were combined. After centrifugation (2500 RPM, 10 min, 4°C), the PBS was removed, and the pellets were again re-suspended in 1 mL ice cold PBS before being centrifuged (2500 RPM, 10 min, 4°C). After removing the PBS, the pellets were re-suspended in 70 μL lysis buffer, vortexed thoroughly, and stored on ice for 15 minutes. The tubes were then centrifuged once more (13200 RPM, 15 min, 4°C) before the samples were transferred to pre-cooled tubes in aliquots (3) of 20 μL and one tube with 10 μL used for measuring protein concentrations. To preserve kinase activity, all microcentrifuge tubes were pre-stored in a -20°C freezer, and tubes with cell lysates were stored on ice during the preparation. Protein concentration was measured with the Pierce^TM^ BCA Protein Assay Kit (cat. no. 23225, ThermoFisher Scientific). The samples were stored at -20°C, and transported to the facility for kinase activity analysis packed in dry ice.

### Kinase activity profiling of human monocytes

Kinase activity profiling using the Tyrosine Kinase PamChip® Array technology (PamGene International B.V., https://www.pamgene.com/en/pamchip.htm) was conducted at the PamStation® 12 platform at Akershus University Hospital, enabling high-throughput profiling of composite kinase activities [29]. The array contains 144 kinase peptide substrates, each consisting of 13 or 14 amino acids with tyrosine residues for phosphorylation (S1 Table). Total soluble protein lysates (above the recommended level >1 μg/μL) from the experimental conditions were incubated on the arrays, using previously described procedures [30]. Total protein (7 μg) was analyzed from all treatment conditions from four biological replicates (four donors) per incubation time, each with three technical replicates. A FITC-conjugated anti-phosphotyrosine antibody was used for visualization during and after the pumping of lysates through the three-dimensional surface of the array. The capture of substrate phosphorylation signals was enabled by a computer-controlled CCD camera and measured repeatedly during a 1-hour kinetic protocol using the Evolve software (PamGene International B.V.). The 1-hour kinetic protocol showed a linear increase in the signal intensity for the majority of the peptide substrates, indicating that kinome profiling was run without methodological complications. With the BioNavigator software (PamGene International B.V.), the endpoint signal intensities generated from bound fluorescent anti-phosphotyrosine antibodies were converted to numerical values. The array data are available in the ArrayExpress data repository www.ebi.ac.uk/arrayexpress) by accession number E-MTAB-4795.

### Data analysis of kinase activity profiles

After visual check and quality control, endpoint signal intensities minus background signals were calculated by BioNavigator for each spot representing each kinase peptide substrate per array. A small number of negative signal intensities were managed by subtracting the 1% quantile of all data and setting remaining signal intensities less than 1 to 1. Subsequently, the data were log_2_-transformed before mean replicate signal intensity within each experiment was calculated for each peptide substrate. One-way analysis of variance (ANOVA) was applied to identify peptide substrates with significantly different phosphorylation levels between experimental conditions. The Dunnett’s many-to-one post-hoc test was applied to adjust for multiple comparisons, and to identify whether experimental conditions significantly altered phosphorylation levels compared with unstimulated controls. Unsupervised hierarchical clustering was performed based on substrate signals’ similarities in treatment response. The clustering is determined by a z-score per substrate which groups peptides with a high correlation in the response pattern to the treatment. This model-based clustering approach used the Bayesian Information Criterion to automatically set the number of clusters.

### mRNA expression data from previously conducted experiments

Previously generated mRNA expression data was uploaded and re-analyzed for the purpose of this study [24]. The data was from an *in vitro* study stimulating elutriated and cryopreserved human monocytes with the experimental conditions summarized in Table 1 [24]. The following experimental conditions were compared for the purpose of this study: 1) monocytes stimulated with *N*. *meningitidis* versus unstimulated monocytes (***Nm vs ctr***); 2) monocytes stimulated with *N*. *meningitidis* in combination with IL-10 versus unstimulated monocytes (***Nm + IL-10 vs ctr***) and; 3) monocytes stimulated with *N*. *meningitidis* and IL-10 versus monocytes stimulated with *N*. *meningitidis* only (***Nm + IL-10 vs Nm***).

**Table 1:** Experimental conditions from which mRNA expression data was generated.

**Experimental conditions**^**a**^	Unstimulated	*N*. *meningitidis* (10^6^/mL)	*N*. *meningitidis* (10^6^/mL) + IL-10 (25 ng/mL)	IL-10 (25 ng/mL)

^a^Human monocytes (10^6^ cells/mL) were stimulated *in vitro* in all experimental conditions

### Ingenuity Pathway Analysis

Gene expression data and kinase activity data was analyzed, and associated figures generated, using QIAGEN’s Ingenuity Pathway Analysis (IPA, www.qiagen.com/ingenuity).

#### Analysis of mRNA levels corresponding to kinase substrates on the PamChip® array

The UniProt accession ID associated with each peptide on the PamChip***®*** array was uploaded to the mapping tool on www.uniprot.org/mapping, which converted the UniProt accession IDs to the corresponding entrez gene IDs. The NetAffx database on www.affymetrix.org was used to identify the probe set ID on the Affymetrix Human Gene 1.0 ST Array. The list of genes corresponding to the peptides on the kinase substrate array was uploaded to IPA. Each gene was loaded to the My Pathway function. Values (FC, *P*-value) from previously generated gene expression data [24] were overlaid in order to identify whether the genes corresponding to array peptides were regulated by *N*. *meningitidis*.

#### Functional analysis, canonical pathway analysis, and identification of molecular relationships using IPA

The array peptides with significantly altered levels of phosphorylation were uploaded to IPA with *P-*values and log_2_-FC. Functional analysis was performed to identify the cellular and molecular functions associated with the array peptides. This analysis is based on expected causal effects between molecules and functions, derived from research findings in the Ingenuity Knowledge Base [31]. Each functional class (such as “Cell-to-cell signaling and interaction”) may have more sub-classes defining more specific cellular and molecular functions (such as “recruitment of mononuclear cells” or “phagocytosis”). The *P*-values and log_2_-FC of the array peptides were used by IPA to predict a decrease or increase in the activity of the function. Significance of the prediction made by IPA is based on a z-score algorithm which communicates the probability that predicted changes in function is a result of chance alone [31]. A z-score above 2 suggests increased activity of the function, while a z-score below –2 suggests decreased activity.

Canonical pathway analysis was performed to identify the pathways significantly associated with the array peptides. The canonical pathways represent delineated biochemical, signal transduction steps for a number of different biological processes. The right-tailed Fisher’s Exact Test was performed to calculate a *P-*value measuring the likelihood that the association between the array peptides and a given function or canonical pathway was due to random chance. A *P-*value <0.05, corrected for multiple testing using the Benjamini-Hochberg method for correcting the false discovery rate [32], was set as threshold for significance of the association.

The list of array peptides was added to “New my pathway” together with pro-inflammatory cytokines known to be released from monocytes after activation by *N*. *meningitidis*. Sub-cellular layout was used to display the molecules according to their primary localization in cellular compartments. The “Build” function was selected, and the tool “Connect” was used to identify molecular relationships between array peptides. The default options were selected for all parameters of the analysis apart from “Confidence level” which was set to include findings that are experimentally observed and highly predicted, and “Tissues and Cell Lines” which was set to include molecular relationships only identified in cells from the immune system.

Finally, another “New my pathway” of the array peptides was created to identify the interactions between these peptides and genes previously shown to be regulated by *N*. *meningitidis* [24]. Specifically, the “Build” function was selected, and the tool “Grow” used to generate these interactions. The default options were selected for all parameters of the analysis apart from: “Confidence level” which was set to include findings that are experimentally observed and highly predicted; “Tissues and Cell Lines” which was set to filter for molecular relationships occurring in cells from the immune system and; “Relationship types” which was limited to transcription and protein-DNA interactions.

### Statistics

One-way ANOVA was used to identify significant differences in mean TNF-release between the experimental conditions. Pairwise post-hoc comparisons using Tukey’s test identified the specific experimental conditions that differed with respect to mean TNF-release. The statistical analysis of the kinase activity profiling data and the gene expression data has been described above.

## Results

### TNF release

**Fig 1:**
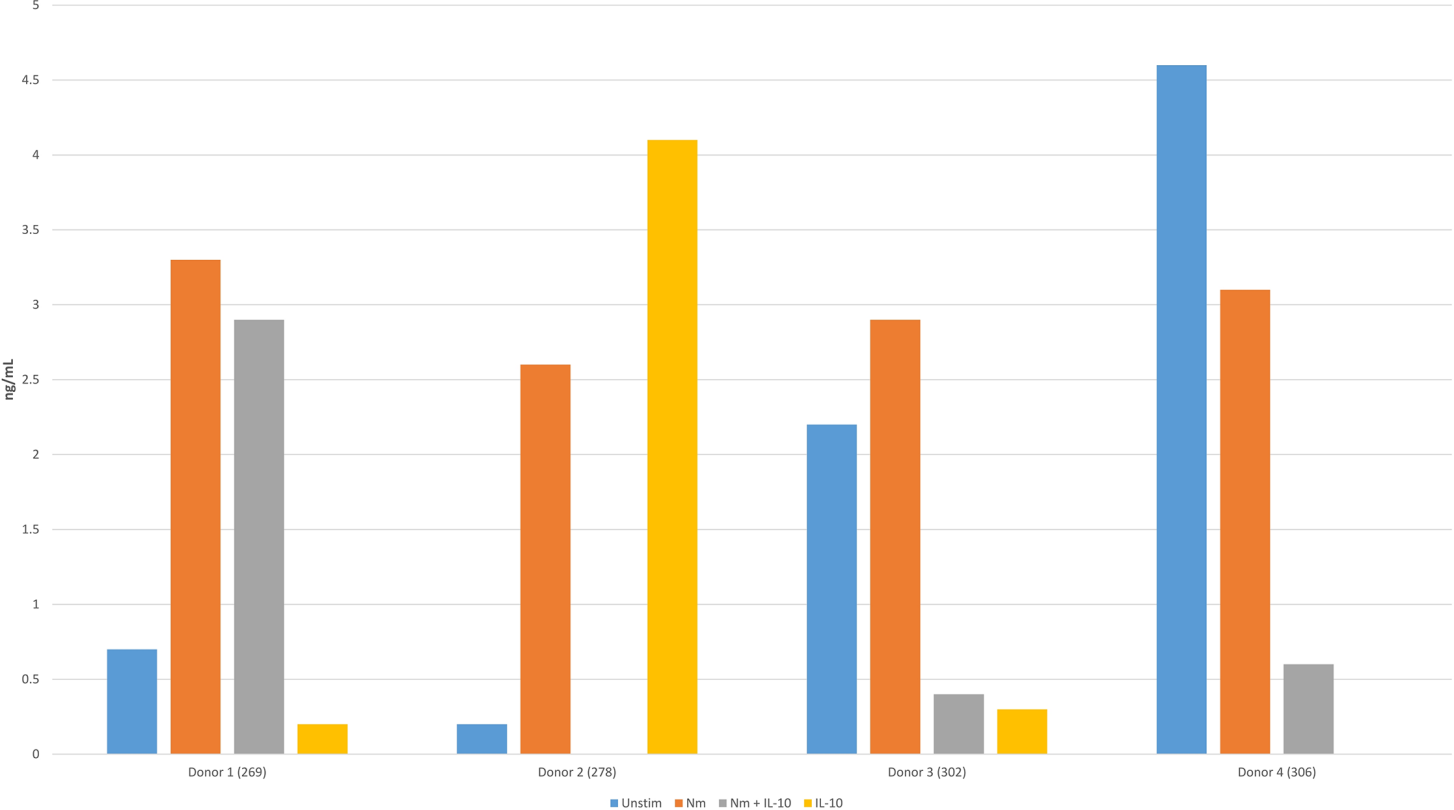
TNF-release (ng/mL) from human monocytes stimulated with *N*. *meningitidis* and IL-10 after 15 minutes.

**Fig 2:**
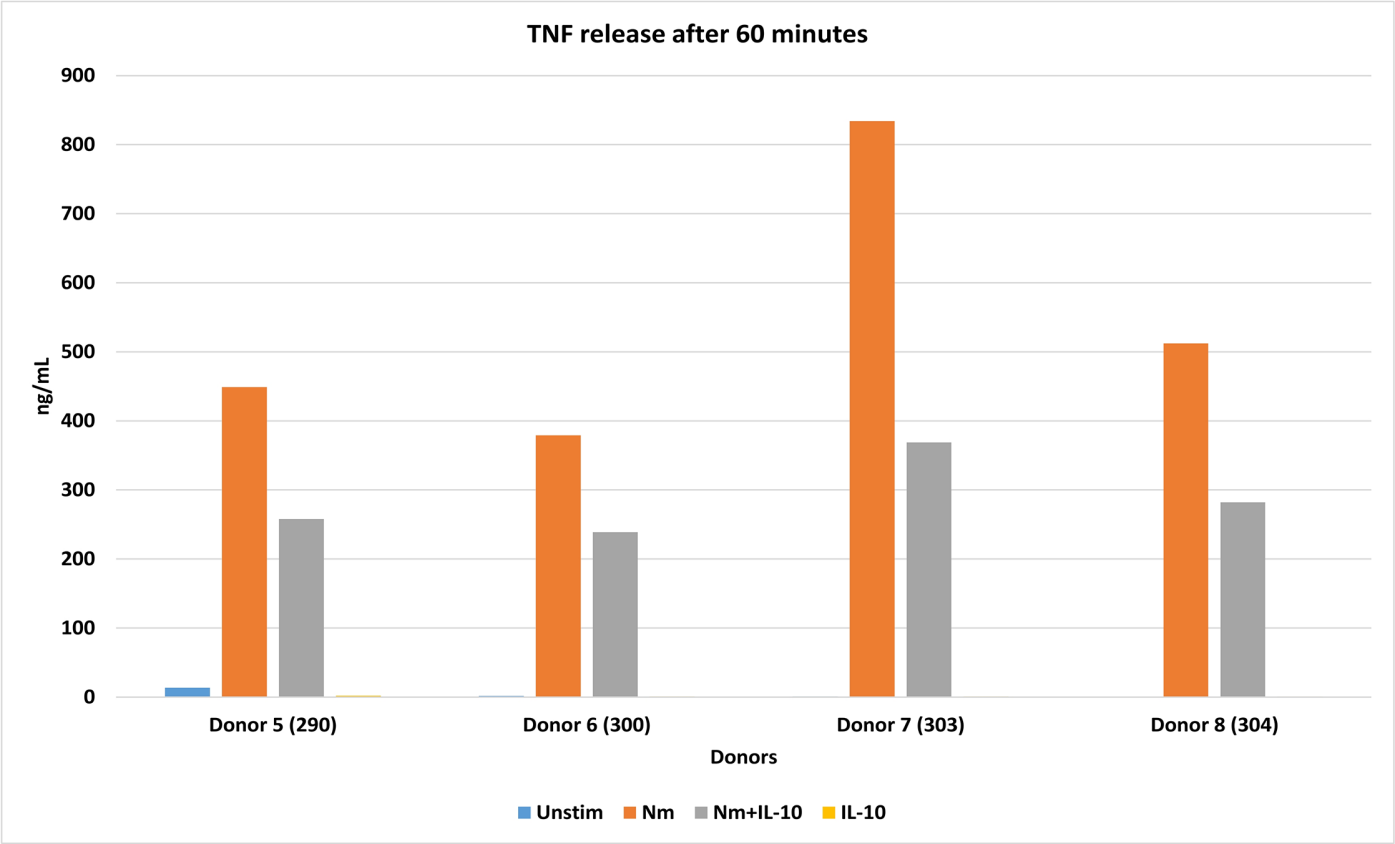
TNF-release (ng/mL) from human monocytes stimulated with *N*. *meningitidis* and IL-10 after 60 minutes. TNF-release after activation of monocytes from donor 303 was checked during an experiment separate from the experiment during which monocyte lysates were generated, due to culture supernatants being discarded by error.

We measured TNF-levels in the supernatants after 15 and 60 minutes to evaluate whether monocytes from the eight donors were activated by *N*. *meningitidis* and inhibited by IL-10 during the incubation (Figs 1 and 2). There were no significant differences in TNF-release between experimental conditions after 15 minutes (*P>0*.*05)*. After 60 minutes, a significant increase (*P<0*.*05*) in TNF from human monocytes stimulated with *N*. *meningitidis* compared with control was detected, consistent with pro-inflammatory activation. When monocytes were stimulated by IL-10 in addition to *N*. *meningitidis*, there was a significant reduction (*P<0*.*05*) in TNF-release (range: 44.9%-36.9%), suggesting that IL-10 induced an anti-inflammatory effect.

### Mapping of mRNA expression levels corresponding to kinase peptide substrates

**Fig 3:**
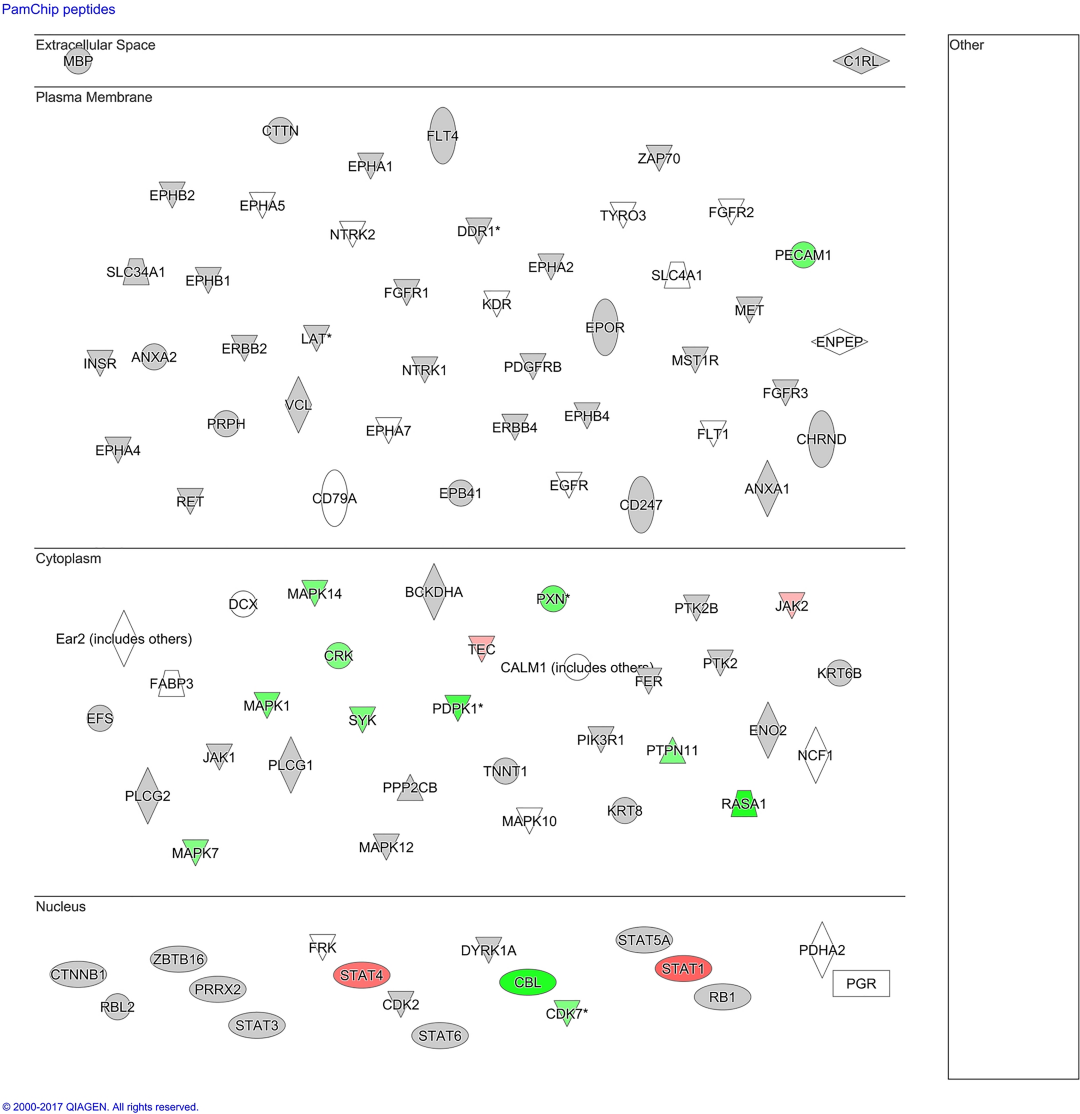
Sub-cellular layout of genes corresponding to peptides present on the kinase substrate array, overlaid with gene expression data from a previous study stimulating human monocytes with *N*. *meningitidis*. The figure shows the sub-cellular location of genes corresponding to peptides present on the kinase substrate array. *P*-values (top value under each molecule) and fold change (bottom value under each molecule) from a previous study comparing gene expression induced by *N*. *meningitidis* with unstimulated controls were overlaid (24). Colored molecules were significantly expressed at FDR 5% in human monocytes stimulated with *N*. *meningitidis* compared with control (a), or in IL-10 immunodepleted plasma compared with low LPS plasma (b). Red indicates up-regulated, green indicates down-regulated.

We wanted a better understanding of whether the peptides on the kinase substrate array were relevant for studying the host response of human monocytes to *N*. *meningitidis*. We therefore used DNA microarray data from our previous study stimulating human monocytes with *N*. *meningitidis* and IL-10 [24], and identified that 19 genes corresponding to these peptides were differentially expressed in monocytes stimulated with *N*. *meningitidis* (Table 2 and Fig 3). Twelve of the 19 genes had reduced expression compared with unstimulated controls, suggesting that meningococci predominantly down-regulated genes corresponding to peptides on the kinase substrate array.

**Table 2:** mRNA expression (fold change) induced in human monocytes by *N*. *meningitidis* and IL-10, corresponding to kinase peptide substrates on the array.

Gene	Experimental conditions^a^		
	Nm vs ctr	Nm + IL-10 vs ctr	Nm + IL-10 vs Nm
CALM1	1.91	1.64	N/C
CBL	-2.85	-2.67	N/C
CDK7	-1.59	N/C	N/C
CRK	-1.57	N/C	1.75
FER	1.75	1.65	N/C
FGFR1	N/C	-1.74	N/C
FLT1	N/C	N/C	N/C
JAK2	1.70	1.74	N/C
MAPK1	-1.84	-1.88	N/C
MAPK14	-1.67	-1.59	N/C
MAPK7	-1.55	N/C	N/C
PECAM1	-1.84	-1.55	N/C
PDPK1	-2.2	-2.15	N/C
PTPN11	-1.60	-1.40	N/C
PTK2B	-1.33	-1.51	N/C
PXN	-1.85	N/C	1.47
RASA1	-2.74	-1.70	1.61
STAT1	3.72	3.04	N/C
STAT3	N/C	1.82	1.69
STAT4	3.3	3.69	N/C
STAT5A	N/C	1.97	1.64
SYK	-1.65	-1.37	N/C
TEC	1.89	1.55	N/C

^a^Statistical significance was set at 5% FDR. N/C = no change (FDR <0.05 and/or FC<1.5).

### Phosphorylation of array peptides by lysates from monocytes treated for 15 and 60 minutes

**Fig 4:**
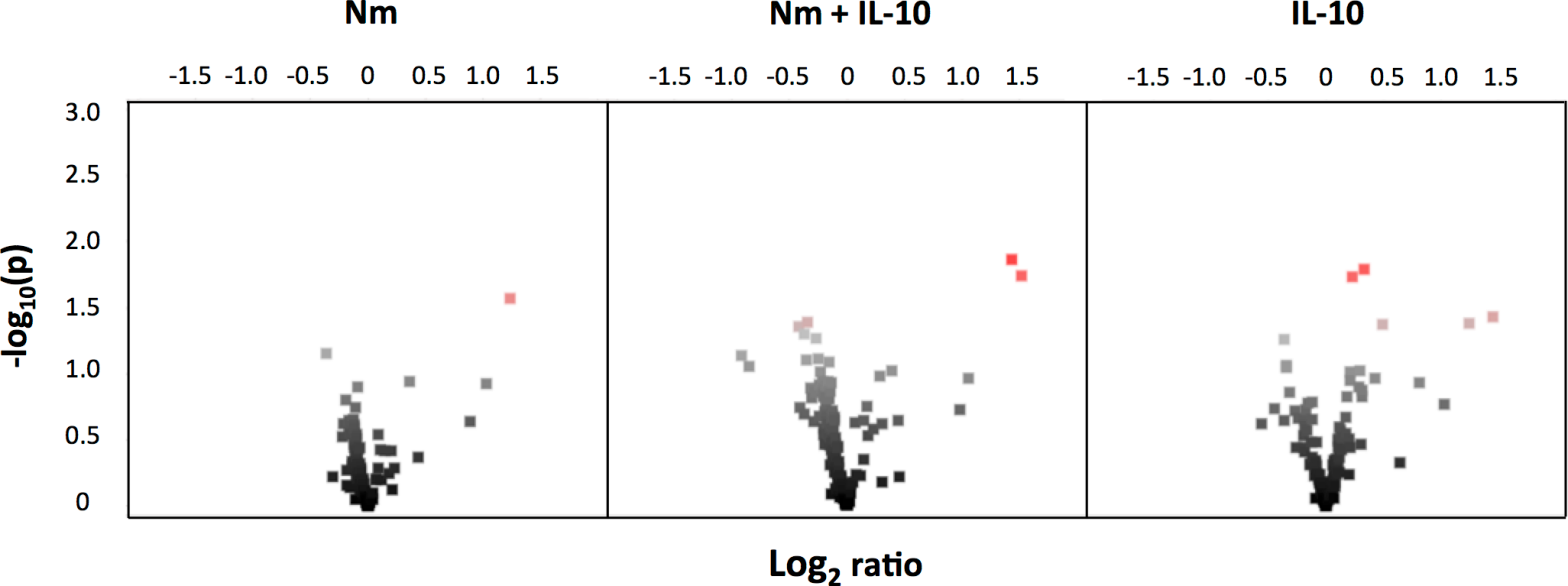
Volcano plot of array peptide phosphorylation after 15 minutes. Log_2_ ratios between treatments (Nm, Nm+IL-10, IL-10) and unstimulated controls. Each dot represents a kinase peptide substrate. Red indicates significantly altered levels of phosphorylation (ANOVA, Dunnet, P<0.05). Dots to the left of the midline of the x-axis represent substrates with decreased phosphorylation, to the right substrates with increased phosphorylation.

**Fig 5:**
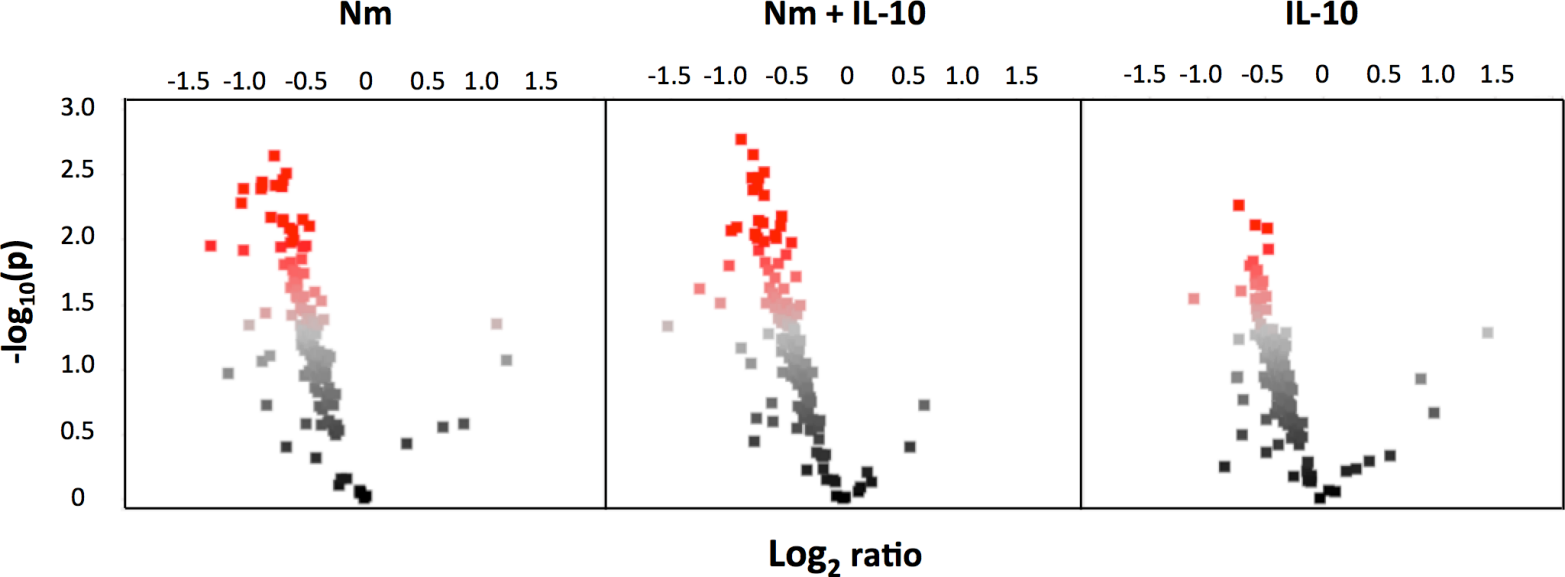
Volcano plot of array peptide phosphorylation after 60 minutes. Log_2_ ratios between treatments (Nm, Nm+IL-10, IL-10) and unstimulated controls. Each dot represents a kinase peptide substrate. Red indicates significantly altered levels of phosphorylation (ANOVA, Dunnet, P<0.05). Dots to the left of the midline of the x-axis represent substrates with decreased phosphorylation, to the right substrates with increased phosphorylation.

**Fig 6:**
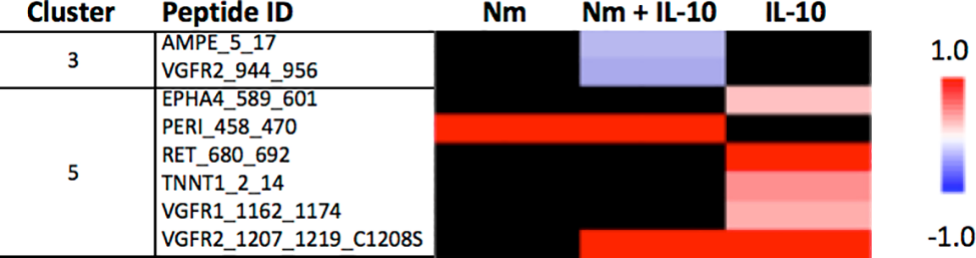
Heatmap of array peptides per cluster with significantly altered levels of phosphorylation following incubation with lysates from monocytes treated for 15 minutes. All treatments (Nm, Nm+IL-10, IL-10) were compared with unstimulated controls. Blue denotes decreased (negative log2 ratio) and red denotes increased (positive log2 ratio) phosphorylation levels. White denotes small, but significant alteration. Black denotes no significant alteration. Unsupervised hierarchical clustering of the kinase peptide substrate phosphorylations was performed based on similarities in treatment response. The peptide ID indicates names of substrates on the array.

**Fig 7:**
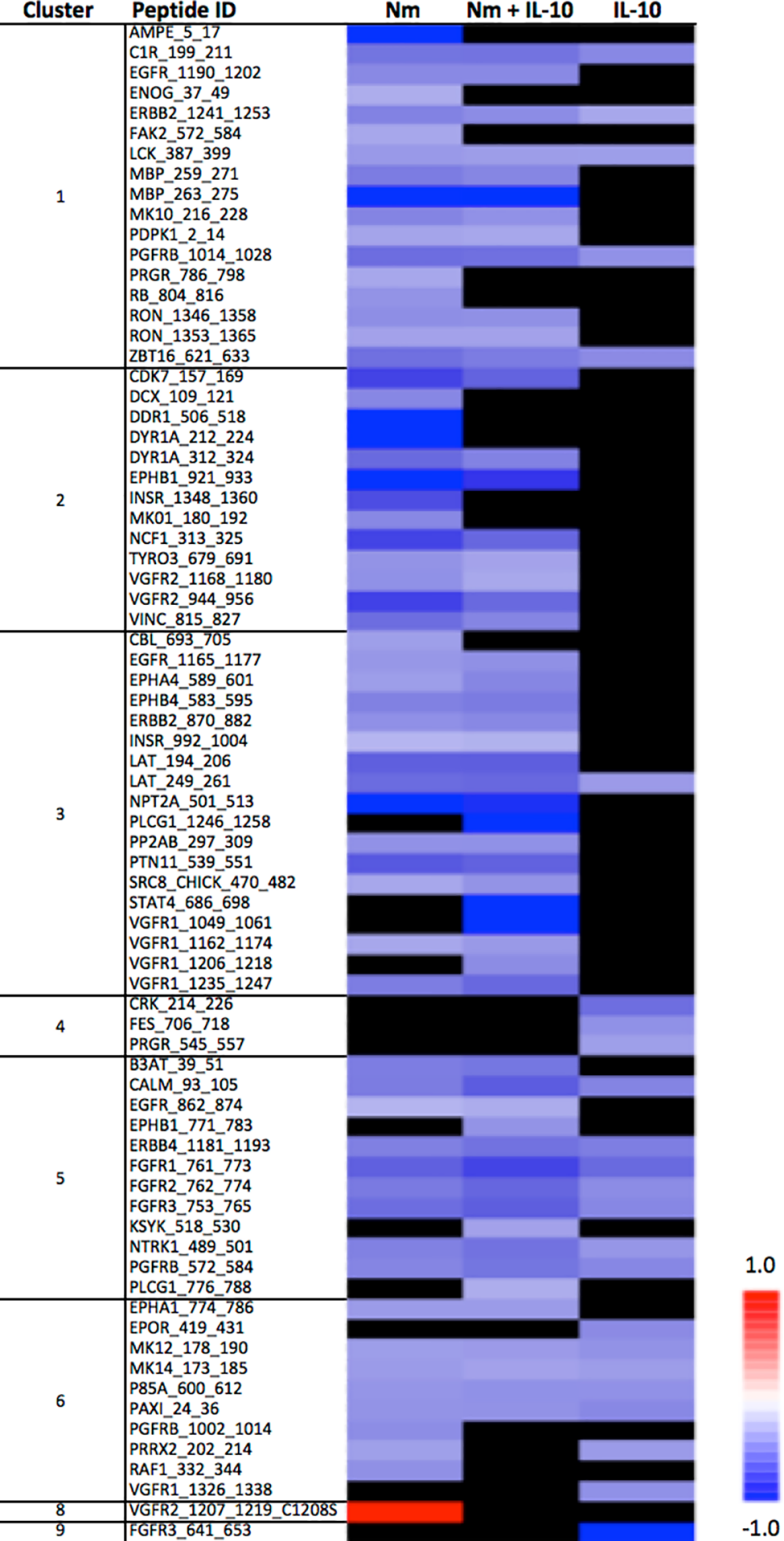
Heatmap of array peptides per cluster with significantly altered levels of phosphorylation following incubation with lysates from monocytes treated for 60 minutes. All treatments (Nm, Nm+IL-10, IL-10) were compared with unstimulated controls. Blue denotes decreased (negative log2 ratio) and red denotes increased (positive log2 ratio) phosphorylation levels. White denotes small, but significant alteration. Black denotes no significant alteration. Unsupervised hierarchical clustering of the kinase peptide substrate phosphorylations was performed based on similarities in treatment response. The peptide ID indicates names of substrates on the array.

**Fig 8:**
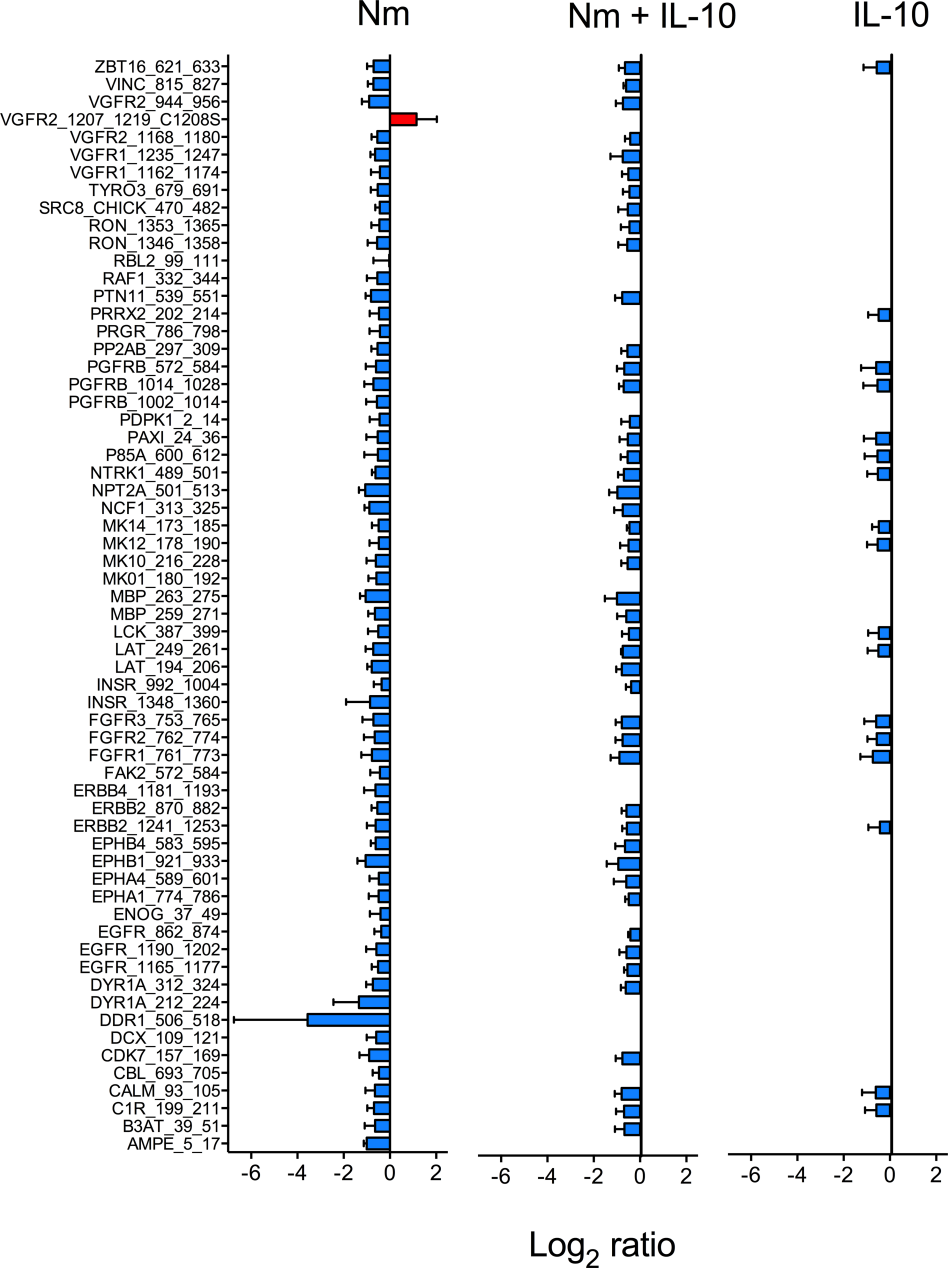
Array peptide phosphorylation induced by lysates from monocytes stimulated with *N*. *meningitidis* (Nm), *N*. *meningitidis* + IL-10 (Nm +IL-10), and IL-10 alone. Blue denotes decreased (negative log2 ratio) and red denotes increased (positive log2 ratio) level of tyrosine phosphorylation. The fig displays 61 kinase peptide substrates identified to have reduced phosphorylation levels when incubated with lysates from monocytes stimulated with *N*. *meningitidis*, compared with lysates from unstimulated monocytes. One peptide substrate (VGFR2) had increased phosphorylation levels. Protein lysates from three biological replicates from each of the three experimental groups (Nm, Nm + IL-10, IL-10) were analyzed to generate the kinase activity profiles. Listed are the substrates’ corresponding gene names in reverse alphabetical order.

We investigated array peptide phosphorylation following incubation with lysates from monocytes treated for 15 and 60 minutes, comparing three experimental conditions (*N*. *meningitidis*, *N*. *meningitidis* + IL-10, and IL-10) with unstimulated controls. After 15 minutes, phosphorylation of only a few peptide substrates was significantly altered compared with unstimulated controls across the three experimental conditions (Fig 4, see S2 Table for *P*-values and log_2_ ratio for all peptides). After 60 minutes, the dominant effect across all three experimental conditions was decreased phosphorylation of array peptide substrates, compared with unstimulated controls (Fig 5, see S3 Table for *P*-values and log2 ratio for all peptides). The kinase substrates with significantly altered phosphorylation level were visualized in a heatmap. After 15 minutes, lysates from monocytes stimulated with *N*. *meningitidis* induced phosphorylation of only one array peptide (PERI, peripherin) (Fig 6). Recombinant IL-10 in turn induced phosphorylation of five array peptides. The significantly altered array peptides after 60 minutes were grouped into nine different clusters based on the similarities in treatment response (Fig 7). Only one array peptide (VGFR2) had significantly increased phosphorylation levels, while the phosphorylation of 61 array peptides were significantly reduced by lysates from monocytes stimulated with *N*. *meningitidis* (Fig 8). Interleukin-10 reduced the phosphorylation of 18 of these 61 array peptides, and reduced in addition the phosphorylation levels of six other array peptides (Fig 8). Lysates from monocytes stimulated with both *N*. *meningitidis* and IL-10 reduced the phosphorylation levels of 54 array peptides (Fig 8). Phosphorylation of 30 of these peptides were reduced by monocytes stimulated with *N*. *meningitidis* alone, while 17 others were reduced by both *N*. *meningitidis* and IL-10 (Fig 8). Statistical analysis comparing array peptide phosphorylation induced by lysates from monocytes stimulated with *N*. *meningitidis* versus monocytes stimulated with *N*. *meningitidis* + IL-10 did not identify the presence of IL-10 to significantly affect changes in array peptide phosphorylation (results not shown).

### Analysis in IPA of array peptides with significantly altered phosphorylation levels

The array peptides with significantly altered phosphorylation by lysates from monocytes treated with *N*. *meningitidis* were selected for functional analysis using IPA (Table 3). The functional categories “cellular development”, “cellular growth and proliferation”, “cellular function and maintenance”, “cell morphology”, and “cellular movement” were associated with the greatest number of array peptides. In IPA’s hierarchy of functions, each higher-level category is composed of more specific cellular functions (known in IPA by the terms “mid-level” and “specific” functions). Within the categories cellular development and cellular growth and proliferation, the array peptides EPHB1, LAT, LCK, MBP, PIK3R1, and PTK2B were associated with reduced cell proliferation of T lymphocytes.

**Table 3:** Molecular and cellular functions associated with the array peptides with significantly altered phosphorylation levels.

Level 1 functional category^a^	Number of molecules associated with the category	Predicted effect on specific functions (activation z-score^b^)	Peptides associated with the specific functions
Cellular development	20	Cell proliferation of T lymphocytes (-2.403)	EPHB1, LAT, LCK, MBP, PIK3R1, PTK2B
		Proliferation of immune cells (-2,040)	EPB1, ERBB2, LAT, LCK, MAPK14, MBP, PIK3R1, PTK2B
Cellular growth and proliferation	19	Cell proliferation of T lymphocytes (-2.403)	EPHB1, LAT, LCK, MBP, PIK3R1, PTK2B
		Proliferation of immune cells (-2,040)	EPB1, ERBB2, LAT, LCK, MAPK14, MBP, PIK3R1, PTK2B
Cellular function and maintenance	17	No specific function significantly affected	
Cell morphology	7	No specific function significantly affected	
Cellular movement	11	No specific function significantly affected	

^a^IPA divides functions into three hierarchical levels: high-level, mid-level, and specific. The table lists the most significant high-level functional categories, and the significant specific functions associated with these.

^b^The activation z-score is a value calculated by the IPA z-score algorithm, to determine the significance of predicted changes, as explained in detail elsewhere [31]. The z-score also predicts the direction of change for the function. A function is predicted to be significantly increased if the z-score is ≥ 2, and significantly decreased if the z-score is ≤ -2.

**Fig 9:**
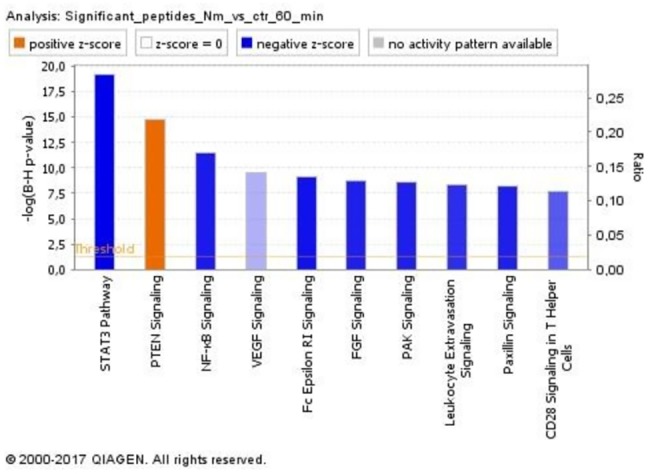
Canonical pathways associated with array peptides with significantly altered phosphorylation. Significantly enriched canonical pathways were identified with a right-tailed Fisher’s Exact Test. The test calculates a *P-*value determining the probability that each canonical pathway associated with the dataset is due to chance alone. A *P*-value of <0.05 was set as threshold for statistical significance. The *P-*values were corrected for multiple testing using the Benjamini-Hochberg method for correcting the false discovery rate. The z-score indicates predicted activation state of the canonical pathway. Blue color or lighter shades of blue indicate a negative z-score and down-regulation of the pathway, and orange color or lighter shades of orange indicate a positive z-score and up-regulation of the pathway. Ratio denotes the number of significantly expressed genes compared with the total number of genes associated with the canonical pathway.

We next identified the array peptides to be associated with alteration of a large number of canonical pathways (top ten displayed in Fig 9). Among the top ten most significantly altered signaling pathways, all but one (PTEN signaling) were predicted by IPA to be inhibited, including STAT3 activation, Nf-κB, and VEGF signaling.

### *In silico* analysis in IPA of links between array peptides and gene expression

**Fig 10:**
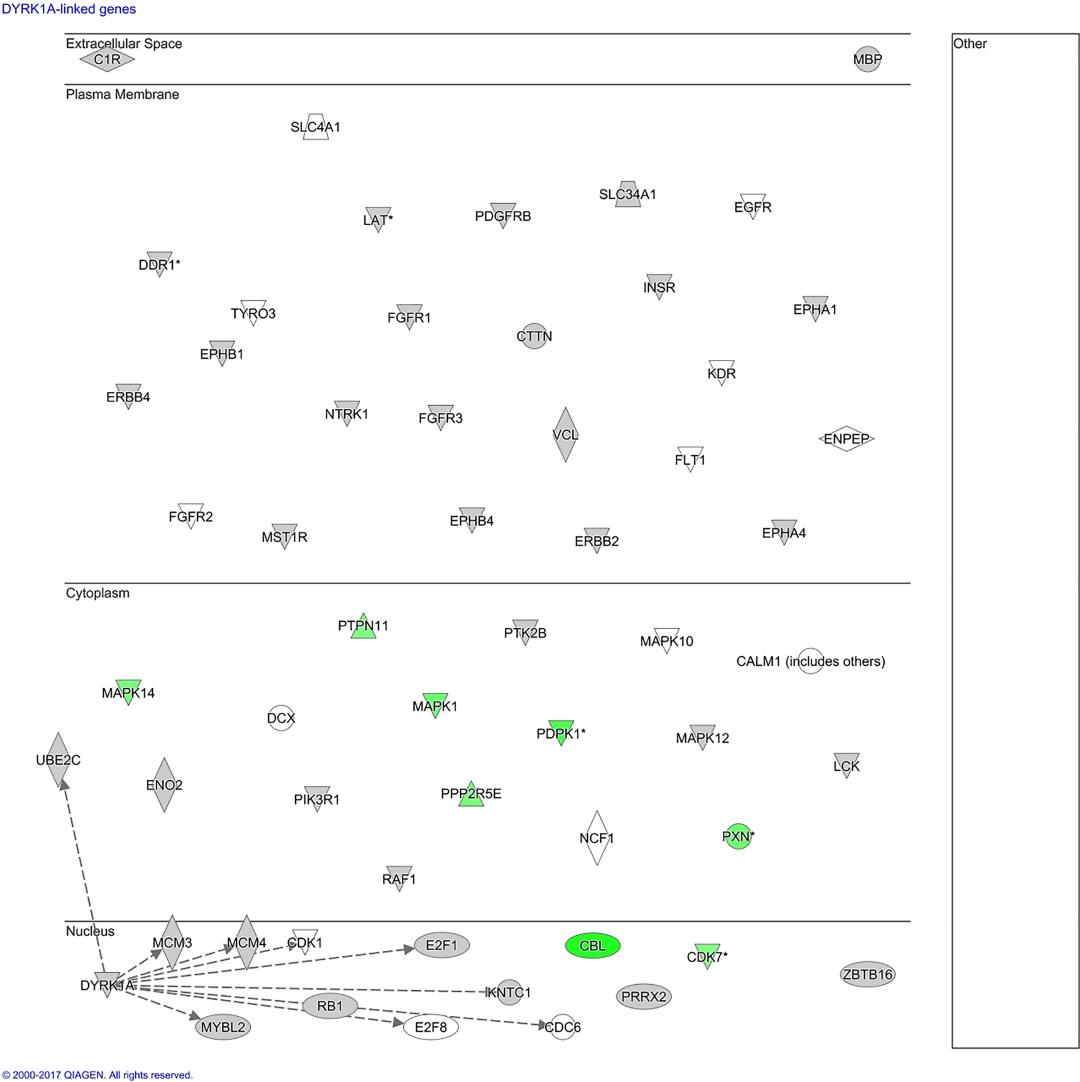
*In silico* analysis of transcriptional activity associated with array peptides with significantly altered phosphorylation levels. The figure displays a sub-cellular layout of array peptides with phosphorylation levels significantly altered by lysates from human monocytes stimulated with *N*. *meningitidis*. IPA was used to identify transcriptional activity of these peptides (see Material and Methods for detailed algorithm). The kinase dual specificity tyrosine-(Y)-phosphorylation-regulated kinase 1A (DYRK1A) was identified to have transcriptional activity linked with the genes CDC6, CDK1, E2F1, E2F8, MCM3, MCM4, MYBL2, KNTC1, and UBEC2. The overlay function in IPA did not identify any of these genes to be regulated by *N*. *meningitidis* (green indicates down-regulation by *N*. *meningitidis*).

We next wanted to identify whether the array peptides might reflect transcriptional activity or protein-DNA interactions by previously identified gene expression changes induced in monocytes by *N*. *meningitidis* [24]. The *in silico* analysis in IPA did not identify any links between the array peptides and genes induced by *N*. *meningitidis* (Fig 10).

### *In silico* analysis in IPA identifies the array peptide ZBTB16 as a regulator of IL-6, TNF, and CXCL10

**Fig 11:**
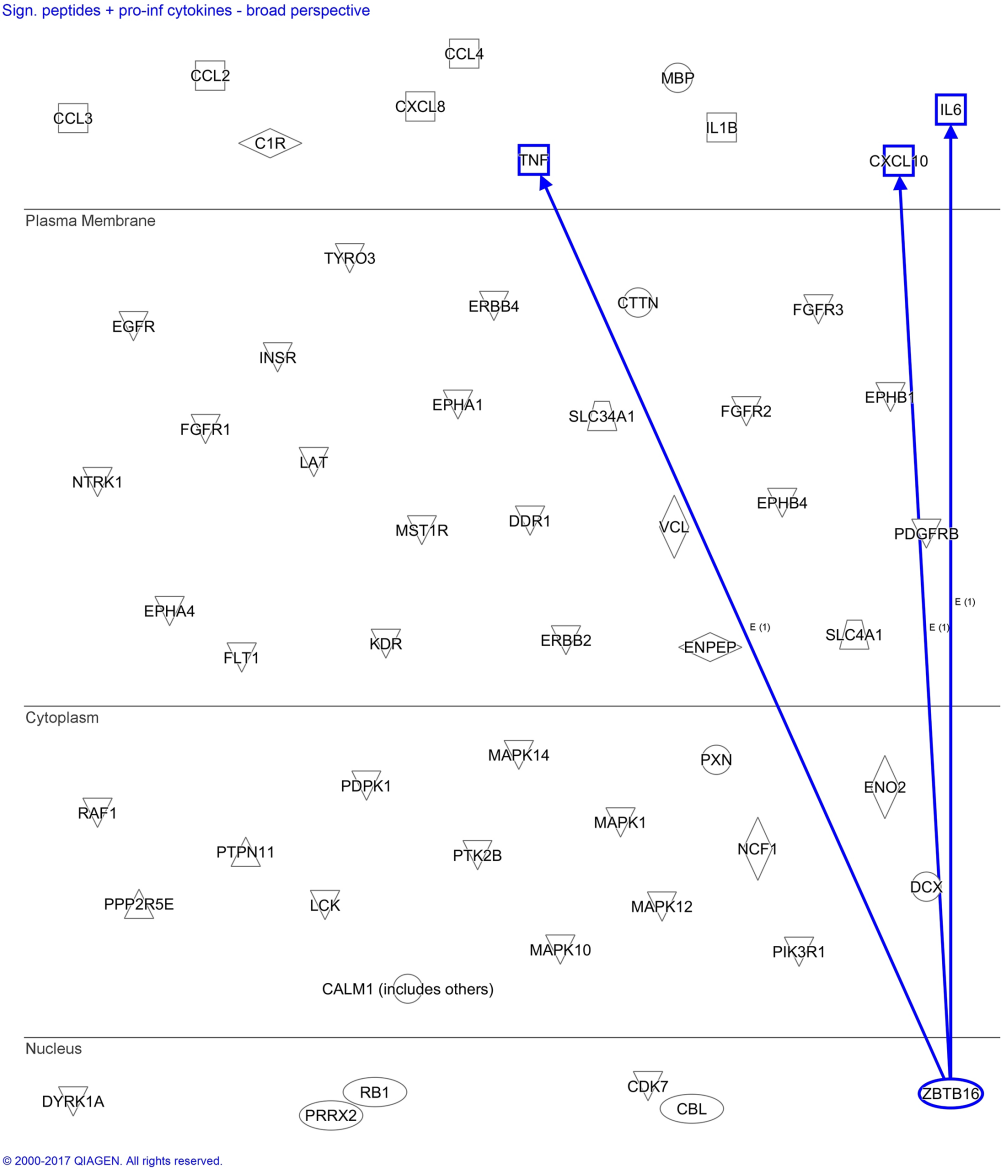
*In silico* analysis of links between array peptides with significantly altered phosphorylation levels and pro-inflammatory cytokines induced by *N*. *meningitidis*. Display of presently known links between array peptides with phosphorylation levels significantly altered by lysates from human monocytes stimulated with *N*. *meningitidis*, and pro-inflammatory cytokines. The pathway analysis tools “Build” and “Connect” were used to identify the links, and connections were filtered to only include direct links between molecules identified in immune cells from human, rat, mice or immune cell line models. *In silico* analysis identified links pointing from ZBTB16 towards TNF, IL-6, and CXCL10.

**Fig 12:**
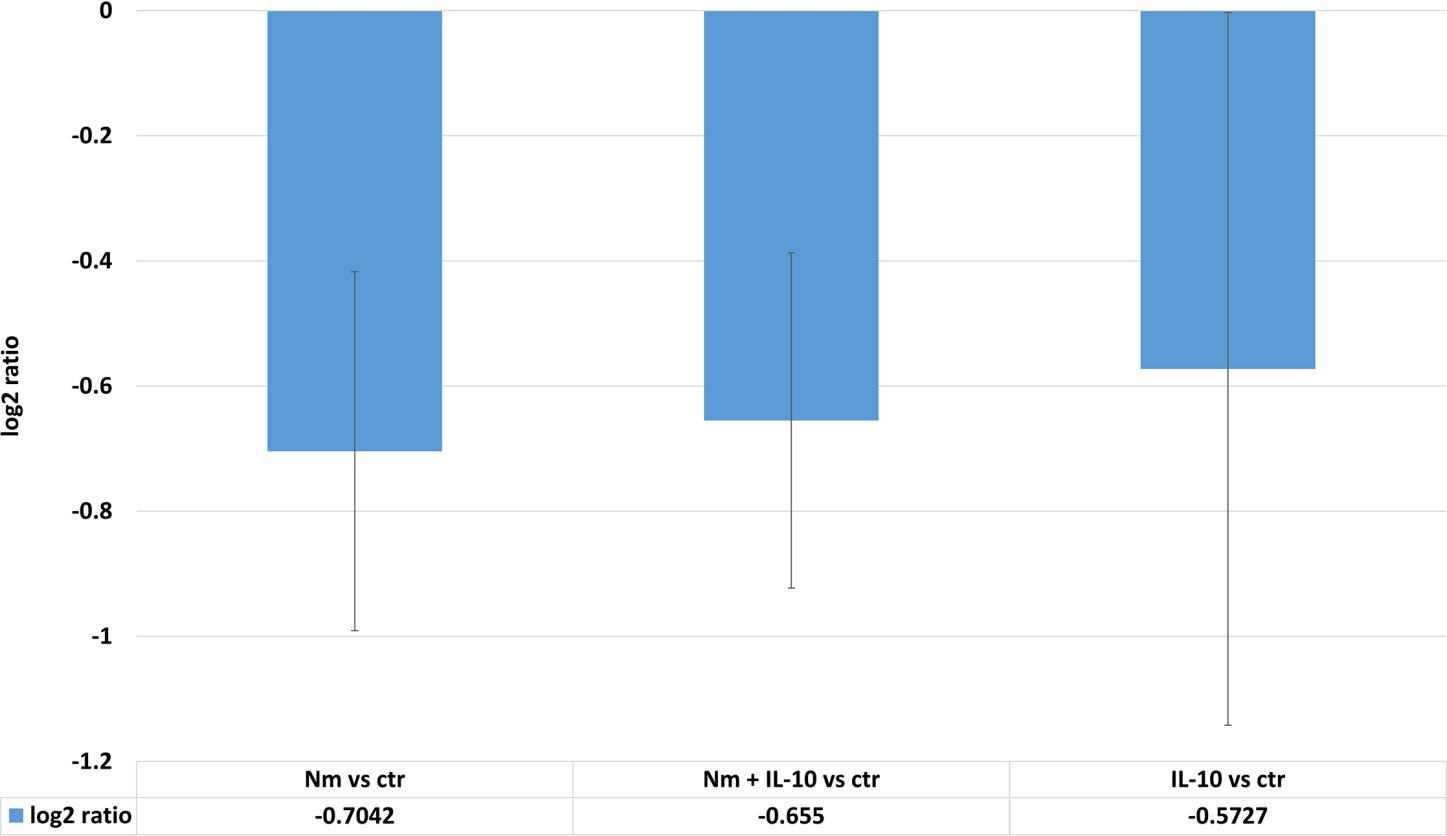
Altered phosphorylation levels (log_2_ ratio) of ZBTB16 by the experimental conditions of this study. Mean log2 ratio calculated by comparison with phosphorylation level in unstimulated monocytes. Error bars denote standard deviation.

We aimed to identify molecular relationships between the array peptides with significantly altered phosphorylation, and a selection of pro-inflammatory cytokines (CXCL10, IL-1β, IL-6, IL-8, MCP-1, MIP-1β, MIP-1α, and TNF) known to be present in large quantities in culture supernatants after monocyte activation by *N*. *meningitidis* [6,9,24]. IPA identified zinc finger and BTB domain containing 16 (ZBTB16), which encodes the transcription regulator known as promyelocytic leukemia zinc finger (PLZF), to be a regulator of TNF, IL-6, and CXCL10 (Fig 11). IPA derived these links from a study showing increased expression of PLZF to be a negative regulator of IL-6, TNF, and CXCL10 [33]. Our kinase substrate analysis located ZBTB16 in cluster 1 (Fig 7) along with 16 other array peptides with reduced phosphorylation by monocytes stimulated with *N*. *meningitidis*. None of the other array peptides were identified by IPA to have links with the selected pro-inflammatory cytokines. The *in silico* finding was validated by the observed reduction in phosphorylation level of ZBTB16 (Tyr630) by monocyte lysates stimulated with *N*. *meningitidis*, *N*. *meningitidis* and IL-10, and IL-10 alone (Fig 12).

## Discussion

Transcriptomic studies conducted in different models such as human endotoxin (LPS) challenge [34–36] of whole blood, purified leukocytes *ex vivo* [10,11,37] and our own *in vitro* studies [5,6,24] have demonstrated the complexity of the host response to sepsis. *Ex vivo* kinase substrate arrays offer an attractive method for parallel analysis of the composite activities of a large number of signaling pathways in order to follow up findings from transcriptomic studies. This present study investigated kinase activities in human monocytes stimulated by *N*. *meningitidis* and IL-10. The first aim was to identify array peptides that could indicate which signaling pathways might be activated or inhibited by the host response to the meningococci. The second aim was to determine whether IL-10 affected *N*. *meningitidis*-induced phosphorylation of array peptides, in order to identify potential targets of the IL-10 anti-inflammatory response. We used an *in vitro* primary cell model of elutriated and cryopreserved human monocytes stimulated with *N*. *meningitidis* and IL-10. The concentrations of these treatments corresponded to previously measured levels in patients with fulminant meningococcal septicemia [4,8,9].

Our study is, to our knowledge, among the first to use the PamChip® tyrosine kinase substrate array technology to investigate LPS-induced pro-inflammatory activation in a human leukocyte population. The technology has primarily been used to identify kinase activity in various cancer types [38,39], and there is a lack of other studies to which our results can be compared. However, already in 2004, Diks et. al [27] conducted kinome profiling in peripheral blood mononuclear cells to study signal transduction by LPS. They used custom designed arrays containing 192 peptides representing substrate consensus sequences across the mammalian kinome. Peak phosphorylation of the main peptides occurred 5–15 minutes after initiation of LPS stimulation, returning to basal levels after 60 minutes of LPS stimulation. Overall, the study concluded that kinome-wide analysis is a promising tool for studying biochemical changes underlying cellular signal transduction. Among the peptides identified by Diks et al. to have increased levels of tyrosine phosphorylation were the substrates PTP-2C, FES, MBP, Annexin-2, and c-raf which are present on the PamChip***®*** array used in our study. Other targets of LPS-induced tyrosine phosphorylation, previously identified by anti-phosphotyrosine immunoblotting techniques, are present on this PamChip***®*** array, including paxillin and Pyk2 (identified in rat peritoneal macrophages [40], and human monocytes and the murine macrophage cell line J774 [26]), Syk (identified in THP-1 cells [41]), and the various mitogen-activated protein kinases, including ERK-2 (identified in rat peritoneal macrophages [42]), JNK3 and p38 MAP kinase (identified in the murine macrophage cell line RAW 264.7 [43]). However, kinome profiling in our experimental model only identified significant increase in phosphorylation of one array peptide by lysates from monocytes treated with *N*. *meningitidis* for 15 minutes. After 60 minutes, a significant decline in kinase activities was caused by monocyte lysates stimulated with *N*. *meningitidis*. The kinase peptide substrates with significantly reduced phosphorylation were associated with inhibition of signaling pathways important to innate immunity, including STAT3 signaling, Nf-κB signaling, and VEGF signaling. This was unexpected given the significant TNF release after 60 minutes, which suggests that our experimental model was activated as expected, and in accordance with our experience with *N*. *meningitidis* as a potent pro-inflammatory activator. However, our previously generated DNA microarray data [24] showed that mRNA corresponding to the array peptides CBL, CDK7, MAPK1, MAPK14, Paxillin, PDPK1, PTPN11, and PP2AB were also reduced by *N*. *meningitidis*, consistent with the direction of change of the phosphorylation of these peptides. The only signaling pathway predicted to be up-regulated was PTEN signaling. This is consistent with inhibition of intracellular signaling, as PTEN has lipid phosphatase activity negatively regulating the phosphatidylinositide 3-kinase (PI3K) pathway, thereby modulating the development and activation of T cells [44]. PTEN signaling has also a central role in the stability of T regulatory cells [45,46], while it is less studied in monocytes. Lipopolysaccharide has been shown to increase expression of PTEN in the fibroblast cell line NIH3T3, but not in the macrophage cell line RAW264.7 [47].

A state of endotoxin tolerance, defined as “reduced responsiveness to a lipopolysaccharide challenge following a first encounter with endotoxin” [48], could have been an alternative explanation for the reduced levels of array peptide phosphorylation by monocytes treated with *N*. *meningitidis*. Several previous studies have indicated that expected phosphorylation following LPS-stimulation is impaired in endotoxin-tolerant cells [49,50]. Our model did not prime human monocytes with LPS prior to the 60 minutes of stimulation, and TNF was detected as expected. This suggests that endotoxin tolerance is an unlikely explanation for the lower levels of array peptide phosphorylation by Nm-stimulated monocytes. Alternatively, it can be speculated whether globally reduced levels of phosphorylation is an acute *in vitro* defense mechanism in human monocytes responding to abrupt and intense activation by large levels of meningococci.

Tyr630-phosphorylation of the peptide representing ZBTB16 (also known as PLZF) was significantly down-regulated by lysates of monocytes after 60 minutes of meningococci stimulation. ZBTB16 belongs to the Broad-complex, Tramtrack and Bric-abrac/poxvirus and zinc finger (BTB/POZ) family of transcription factors. *In silico* analysis identified ZBTB16 to have direct links with IL-6, TNF, and CXCL10. This finding in IPA is derived from a recent study that showed bone-marrow derived macrophages from ZTBTB16-/- mice to have increased mRNA expression and cytokine release of IL-6, TNF, and CXCL10 [33]. Chromatin modification, control of NF-κB and histone deacetylase activity was suggested as mechanisms by which PLZF repress IL-6, TNF and CXCL10. To our knowledge, it is not currently known whether phosphorylation of PLZF is required for its repressing effect on IL-6, TNF and CXCL10. The specific kinases that phosphorylate PLZF have not been identified by previous studies of PLZF in innate immunity [33,51].A hypothesis that emerges from our *in silico* analysis is whether reduced phosphorylation of PLZF by LPS results in reduced activity of PLZF, with subsequent increased release of IL-6 and TNF, and mRNA expression of CXCL10. However, we also found IL-10 to significantly reduce phosphorylation levels of ZBTB16, which biologically would be inconsistent with known anti-inflammatory effects of IL-10 on these cytokines. Overall, there is uncertainty about the role of reduced ZBTB16 phosphorylation in the pathway leading to increased levels of IL-6, TNF, and CXCL10. Existing anti-phospho antibodies target Tyr334, and not Tyr630, which is the residue present on the array peptide, and it has at present not been possible to further validate an association between reduced tyr630 phosphorylation of ZBTB16, and reduced cytokines levels of IL-6, TNF and CXCL10. Additional experimental validation, for example by measuring TNF, IL-6, and CXCL10 after implementing methods for overexpressing ZBTB16 in LPS- and IL-10-stimulated cells, is also needed to test the effects of ZBTB16 on Nm- and IL-10-induced release of these cytokines.

Decreased levels of LPS-induced tyrosine phosphorylation has for long been suggested as a mechanism by which IL-10 mediates its anti-inflammatory response [28,52,53]. However, our kinome profiling did not identify significant differences in array peptide phosphorylation between *N*. *meningitidis*-stimulated monocytes and monocytes stimulated with *N*. *meningitidis* and IL-10. Our study was therefore unsuccessful in identifying potential targets for the IL-10 anti-inflammatory response, which we previously have studied at the gene expression (8,20) and cytokine level [6,7]. Whether IL-10 at higher levels or incubated with *N*. *meningitidis* for longer incubation times can alter LPS-induced kinase activity remains to be investigated.

## Conclusion

In our experimental model of activating human monocytes with *N*. *meningitidis* for 15 and 60 minutes respectively, kinome profiling suggested a profound inhibitory effect in terms of *ex vivo* array peptide phosphorylation by monocytes treated for 60 minutes. This suggests decreased activity of a large number of kinases associated with different signaling pathways and immune cell functions. A hypothesis emerging from the *in silico* analysis is whether reduced phosphorylation of ZBTB16 is a mechanism by which meningococcal LPS contributes to increased expression of TNF, IL-6, and CXCL10. In our experimental model, kinome profiling was not able to detect a statistically significant increase in early (after 15 minutes of monocyte treatment) array peptide phosphorylation of previously known targets of LPS-signaling. Targets of the IL-10 anti-inflammatory response were not identified. Further studies should investigate changes in tyrosine kinase-mediated signal transduction in human immune cells, in order to evaluate the potential clinical application of the kinase substrate array in the study of the systemic inflammatory response to pathogens.

## Supporting information

S1 Table Overview of peptides on the tyrosine kinase array. (XLS)

S2 Table P-values and log_2_ ratio for all peptides after 15 minutes. (XLSX)

S3 Table P-values and log_2_ ratio for all peptides after 60 minutes. (XLSX)
